# A Behavioral Economic Characterization of the Impact of the COVID-19 Quarantine on Reinforcer Diversity, Behavior, and Health

**DOI:** 10.1007/s40614-026-00502-7

**Published:** 2026-05-15

**Authors:** Erin B. Rasmussen, Alam Y. Alvarado

**Affiliations:** Department of Psychology, Idaho State University, Pocatello, ID USA

**Keywords:** Alternative reinforcement, Behavioral economics, COVID-19, Health, Isolation, Quarantine, Reinforcer diversity

## Abstract

In 2020, the U.S. government, like other countries, instated a nationwide quarantine to reduce the spread of COVID-19, which had reached pandemic status. Though challenging on a number of levels, the quarantine also provided a unique opportunity to examine the effects of a cultural phenomenon that reduced access to many sources of reinforcement and the potential impact on behavior and health. From a behavioral economic standpoint, the quarantine likely resulted in greater consumption of reinforcers that were highly available in the home, such as social media, sleep, substances, and food, while lowering consumption of reinforcers that were restricted by quarantine policies. These two trends likely combined into a net reduction in the diversity of reinforcers that were available to individuals during the quarantine. For some individuals, these shifts in consumption also predicted changes in health and mental health status, especially those involving consumption of social media, sleep, substances, and food. We discuss the implications for health and mental health associated with these trends and end by offering suggestions on how a more careful programming of available reinforcers during times of low reinforcer diversity may prevent excessive consumption of reinforcers that may affect health or mental health risks.

In many situations, access to a wide array of reinforcers may abruptly be reduced. On an individual level, this may include what occurs during a time-out—a practice that decreases a targeted unwanted behavior. A time-out involves brief removal of an individual (usually a child) from an environment that includes a range of reinforcing stimuli, such as toys, games, or other people (e.g., see reviews Dadds & Tully, 2019; Knight et al., 2020). Another example of a sudden reduction in reinforcement might occur with hospitalized patients as they undergo and heal from a procedure or injury. Such instances may result in acute or long-term restrictions from one’s typical variety of reinforcers. These restrictions may come not only from physical limitations in mobility, but also from a sterile recovery room that has few of the reinforcers from a person’s everyday interactions at home, work, school, or hobbies. This is likely what makes hospitalizations aversive for adults and children (Koukourikos et al., 2015). Likewise, older patients limited by mobility or moved to nursing homes may have limited opportunities for activity or interaction with others; these individuals often report feelings of isolation, loneliness, and poor quality of life (Pitkala, 2016). Other forms of institutionalization in residential settings may also restrict sources of reinforcement. Incarcerated individuals, for instance, must transition from a community with a wide range of reinforcers (e.g., employment, social interactions, recreational activities, and personal freedoms) to an environment that is restricted from these activities, stimuli, and events. Indeed, this type of penalty is implemented to deter or reduce criminal behavior.

There are also abrupt losses of reinforcement that occur at the sociocultural level that affect larger groups or even entire populations of people. One example is a localized (e.g., statewide or community-based), nationwide, or global quarantine. Quarantine refers to a restriction of movement and interaction of people (or nonhumans) who may have a disease or virus to reduce further transmission. Nationwide quarantines were implemented across the globe in 2020 to reduce the spread of COVID-19—a moderate to severe respiratory virus that was responsible for over 7 million deaths worldwide (WHO, 2025). In the United States, COVID-19 quarantine policy and practices varied by state, and also at the level of cities and townships. Despite these variations, however, quarantine measures implemented early in the pandemic slowed the spread of the virus, thereby saving lives (Allcott et al., 2025).

The COVID-19 quarantine period also provided a unique opportunity to examine the collective and individual effects of a broad sociocultural event on behavior, and in particular, how abrupt changes in access to a variety of reinforcers may influence behavior and mental health. This article uses the framework of behavioral economics to examine how the constraint of reinforcement during quarantine predicted changes in the consumption of reinforcers with varying levels of availability. A culture-wide quarantine might predict increases in consumption of highly available reinforcers, as well as reductions in more restricted reinforcers, leading to a net reduction in *reinforcer diversity*. We introduce the term of reinforcer diversity briefly here as the number and quality of reinforcers that are typically contacted in a given period of time for an individual, though the concept is explicated in more detail later in this article. We also integrate literature from public health and clinical psychology with behavioral economics and offer some of our own data to describe these behavioral trends in consumption and relate them to health and mental health change during the COVID-19 quarantine. In doing so, we identify what we believe is a common mechanism among these literatures in predicting mental health and health outcomes—abrupt restriction in the range and diversity of reinforcement.

## A Brief Characterization of the COVID-19 Pandemic and Quarantine Measures

It is important to first describe the nature of the pandemic and why quarantine was implemented. In 2019, SARS-CoV-2, a highly contagious acute virus that causes COVID-19 was identified in China’s Hubei Province (Narayanan et al., 2024). It quickly spread through Asia and then to over 200 other countries, mostly via cruise ship and air travel, where it became an extreme public health threat (Ahmadi et al., 2020). During this time period, concern for the spread of the virus grew as symptoms for COVID-19 varied from mild (e.g., fevers, coughs, and loss of taste) to severe (e.g., difficulty breathing, persistent chest pains, and skin discoloration), with the latter possibly leading to hospitalization or even death (Centers for Disease Control & Prevention [CDC], 2024b, 2024c). It is also important to note that some individuals who tested positive for the virus were asymptomatic, further supporting the variation in sensitivity and symptomology of the virus. Nonetheless, the virus was deadly for some. The World Health Organization (WHO) estimates that nearly 3 million people died worldwide in 2020 alone (WHO, 2021), though by late 2025 (the time this article was written), over 7 million COVID-related deaths have occurred (WHO, 2025).

The quarantine period, which was implemented early in the pandemic, allowed scientists to determine and ascertain the viral transmission mechanisms, so that more refined measures could be implemented to reduce contagion. During this time, it was found that COVID-19, and its later variants (e.g., alpha, beta, gamma, and omicron) are transmitted through airborne molecules (e.g., sneezing, coughing, and talking) and travel up to 6 feet and may remain suspended in the air for several hours (CDC, 2024a). It was also determined that older individuals and those with respiratory health conditions were more susceptible to the more severe symptoms. Therefore, masks, hygiene, and social distancing (i.e., maintaining at least a 6-foot distance from others) was recommended by the CDC.

As mentioned, quarantine measures varied widely by country, and in the United States, they also varied by state. In the United States there were some common practices for most of the country. In the early part of the quarantine, nonessential businesses (e.g., those generally for recreation and entertainment, such as bars, salons, restaurants, and large venues) and schools were required to temporarily close, and people were encouraged to stay inside their homes, reducing social interaction. The duration for quarantine periods varied widely by population and geographical area. For example, in New York City, a densely populated area, in-person public schools were closed in March 2020, and reopened a year later in March 2021. However, online and hybrid (a combination of in-person and online) schooling formats were implemented for K–12 and university classes. On the other hand, in the less populated state of Idaho, the governor issued a stay-at-home order in late March 2020, which ended one month later; however, nonessential businesses remained under “lockdown” until mid-June 2020 (Heyward, 2021; Hyer, 2020). During quarantine and after quarantine ended, the Centers for Disease Control (CDC) continued to encourage people to wear masks and practice social distancing, and when later developed, the COVID-19 vaccine was recommended to effectively reduce transmission and risks.

Though the quarantine reduced the transmission of COVID-19, it was not without consequence. There was a great deal of public outcry about quarantine measures in the United States. Some of the concern was political (e.g., see Caceres et al., 2022; Roozenbeek et al., 2020), whereas others were related to economic threats of closing businesses and unemployment, which resulted in direct income loss for many (Anyamele et al., 2022; Kannan & Khan, 2022), as well as a cascade effect that affected supply chain processes by way of reducing production, storage, distribution, and transportation of goods. This disruption resulted in economic contraction. Although some businesses were able to adapt to the quarantine measures (e.g., some dine-in restaurants moved to a to-go format only), others were not able to remain open. Despite government efforts to provide economic and nutritional assistance, income and food security status of many families were negatively affected (e.g., Fang et al., 2022). Some office-types of businesses were able to convert to remote format, which allowed them to remain open, but this also created a host of its own problems, such as stressors involved with working from home environment while caring for children who were also learning remotely from home (Deeb et al., 2022; Giannotti et al., 2022; Toran et al., 2021).

An additional quarantine-related concern was mental health related to social isolation. Many were concerned that staying home for most of their day might affect mental health. Children, for instance, may have restricted access to effective ways of learning at school and opportunities for social interactions with peers and friends (e.g., participation in sports events and after-school extracurricular clubs). These types of activities have been shown to be important to child well-being, especially to those who have lower income or are from marginalized groups (Hawrilenko et al., 2021).

### Social Isolation and Health

The concerns about quarantine and social isolation were likely valid ones. A great deal of research has been conducted on associations between social isolation and mental health. Social isolation is defined as disengagement from social ties, institutional connections, or community participation, and indeed may be considered a form of extinction or removal from sources of social reinforcement. Social isolation may have long-lasting effects on the mental health of children and adults, including anxiety and depression (Cacioppo et al., 2006; Loades et al., 2020; Seeman, 1996). Moreover, individuals may feel loneliness (i.e., the subjective disconnection from others) as a result of social isolation, which is also associated with increased risk of mental health problems. The effects of social isolation were studied as early as 1979 in which chronically socially isolated adults had higher rates of mortality compared to less socially isolated adults (Berkman & Syme, 1979; House et al., 1988). Individuals who are isolated or report higher feelings of loneliness are at higher risk of depression, suicide, and even dementia (Leigh-Hunt et al., 2017; Matthews et al., 2016; Teo et al., 2013; Wolters et al., 2023).

Not only are individuals who are socially isolated at risk for mental health problems, they are also at risk for engaging in substance use. Research has shown that teens who are socially disconnected from their peers have greater health risks than socially connected teens (Copeland et al., 2018). For example, isolated teens are more likely to use substances to manage self-reported feelings of loneliness and anxiety (Heinrich & Gullone, 2006; Kobus & Henry, 2010; Osgood et al., 2014). As a result, these individuals are at greater risk for developing substance use disorders and long-term health problems, such as cardiovascular disease and cancer (National Institute on Drug Abuse, 2020).

Alcohol Use Disorder (AUD) is a chronic psychological disorder that is attributed to the deaths of 175,000 people annually in the United States and 3 million people worldwide (King et al., 2025). Research suggests a bidirectional relation between social isolation and an increased risk of AUD in which individuals who are socially isolated are at higher risk of engaging in alcohol consumption, depression, and suicide (Clay et al., 2023; Walsh & Schlauch, 2024). Further supporting this trend are nonhuman experimental studies, which show that rodents that are socially isolated after weaning also show elevated alcohol consumption during adulthood compared to those that are not socially isolated (Butler et al., 2014; Lesscher et al., 2015).

Social isolation is also linked to higher consumption of food (see review by Hanna et al., 2023). Some research shows that individuals who are socially isolated or lonely may eat to “fill a void” (Ljubičić et al., 2023; Troisi & Gabriel, 2011). Indeed, research in neuroscience shows that common structures that are activated by an absence of social interaction are also tied to neural substrates that occasion humans to search for and eat food (see Cacioppo & Patrick, 2008). In addition, depression, which is a chronic state in which anhedonia, or lack of sensitivity to reward or reinforcement, can be characterized by low social interaction and higher food intake (see Epstein et al., 2007a, 2007b); indeed, this reciprocal relation can spiral into weight gain that compromises long-term health. These research areas, as a whole, show that chronic loneliness, by way of low access or sensitivity to social reinforcement, is also tied to increases in food intake and a greater risk of food-related health concerns, such as obesity.

Thus, from a health and mental health standpoint, social isolation is related to several health and mental health problems, and some of these involve the consumption of primary reinforcers, such as substances and food. Social connection is critically important to most individuals and has evolutionary ties to survival (Roberts et al., 2014). Indeed, social relationships are so important to most humans, that when rejected from a social group, brain regions that activate in response to physically painful stimuli are stimulated (Eisenberger, 2012a, 2012b). Stated another way, rejection and isolation for many may subjectively feel physically painful.

From a behavioral standpoint, social connections can be regarded as reinforcers; the presence of social consequences (e.g., speaking kindly, smiles, laughter, physical contact) of certain individuals over others is what reinforces the behavior of seeking, approaching, and communicating with them (e.g., Jones et al., 2011; Vollmer & Hackenberg, 2001). Indeed, social isolation is a situation in which access to social reinforcers, especially by those we prefer or value, diminishes abruptly or gradually. A pandemic that restricts movement and interaction of a body of people would presumably restrict social reinforcement, in addition to other sources of reinforcement, causing a redistribution of behavior to available reinforcers. A theoretical lens that seems relevant to such an analysis is the behavioral economic framework.

## Behavioral Economics and Quarantine

Behavior economics is a field of study that incorporates principles from economics and the laboratory rigor of the experimental analysis of behavior to understand choice behavior (Hursh et al., 1988; Hursh, 1980; Hursh, 1984; Madden, 2000; Mullainathan & Thaler, 2000). The behavioral economic paradigm predicts that the consumption of a reinforcer is related to its availability as well as the availability of other competing reinforcers (Hursh et al., 1988; Hursh, 1980; Hursh, 1984; Madden, 2000; Mullainathan & Thaler, 2000). Some aspects of availability that are well-established in determining choice for one reinforcer over others include the relative densities and qualities of reinforcers that are available. In addition, the effort and delay involved in accessing these varying sources of reinforcement are important conditions. These are briefly described below.

First, the *amount* of reinforcement that is derived from one option, relative to other options, is one factor in behavioral allocation. For example, if twice the amount of reinforcement is available from option A compared to option B, roughly twice as much behavior is likely to be allocated to option A compared to option B; this is often a proportional and linear relation (i.e., the matching relation; Herrnstein, 1974; Baum, 1974). For instance, consider when two sources of the same food are available under concurrent schedules of variable interval reinforcement that arrange a 5:1 or 1:1 ratio of reinforcement on two levers (e.g., Buckley & Rasmussen, 2012; Buckley & Rasmussen, 2014). When the ratio is 5:1, rats will allocate roughly five times more behavior to the richer source of reinforcement. When the ratio is 1:1, they will allocate roughly half of their behavior to each lever. This ability to “match” behavioral allocation to the relative amount of reinforcement has been shown for decades across many species including pigeons, humans, rodents, fish, bees, and primates and is considered a general principle of behavior (e.g., Banna et al., 2011; Buckley & Rasmussen, 2012; Herrnstein, 1974; Iigaya et al., 2019; Rasmussen & Newland, 2008; see Davison & McCarthy, 2016, for review). In addition, the matching relation holds in applied contexts, such as with the behavior of individuals with intellectual disabilities. For instance, using the matching relation, Borrero and Vollmer (2002) replaced severely problematic behavior (e.g., aggression and self-injury) with more appropriate behaviors, such as requests for attention or objects (Borrero & Vollmer, 2002). The matching relation even predicts behavior in professional sports, including football (Reed et al., 2006), baseball (Cox et al., 2017; Falligant et al., 2021), and basketball (Vollmer & Bourret, 2000).

Relative amount is important, but so is reinforcer quality (e.g., Sinha et al., 2019). For example, if an organism is choosing among different available foods, palatability, often determined by sugar content or fat content, may result in higher behavioral allocation than foods that are less palatable. This occurs regardless of relative amount and can be measured quantitatively as bias or hedonics (Baum, 1974; Boggiano et al., 2009; Buckley & Rasmussen, 2012; Herrnstein, 1974; Miller, 1976). For instance, in a study by Buckley and Rasmussen (2012), when controlling for relative amount, rats, especially obese ones, showed strong preferences for sucrose over grain pellets, often forgoing relative amount for palatability.

In addition, price, by way of effort and money (a fundamental variable in macroeconomics) also play a role in consumption of a reinforcer, in that greater prices reduce consumption of reinforcement (Epstein et al., 2000; Epstein et al., 2010; Epstein et al, 2018; Hursh et al., 1988; Hursh et al., 2005; Hursh & Silberberg, 2008; Rasmussen et al., 2012; Rasmussen et al., 2016). In a choice context, if a reinforcer requires more effort to access, it is less likely to be chosen over other similar reinforcers that are less effortful (e.g., Botvinick et al., 2009; Białaszek et al., 2017; Bickel & Vuchinich, 2000; Carroll et al., 1989; see Salamone et al., 2009; Salamone et al., 2018). For instance, in a T-maze, if the two choice arms contain a similar amount of food (e.g., two pellets), the arms will be preferred equally. If a physical barrier is placed in front of the food available in one of the arms, the one without the barrier is preferred. Indeed, it may take twice the number of pellets (e.g., 4 pellets) to compete for behavioral allocation relative to the 2-pellet option with no barrier (e.g., see Salamone et al., 1994, for basic procedure). Increasing response cost has also been applied to a large number of behaviors using applied behavior analysis (see reviews by Friman & Poling, 1995, and Wilder et al., 2021). Some of these include the reduction of problematic behavior, such as self-injurious behavior (e.g., Fisher et al., 1997; Morgan et al., 2017) and pica (e.g., Piazza et al., 2002). Others include increasing behaviors involved in recycling (e.g., Fritz et al., 2017; Miller et al., 2016) and occupational safety (e.g., Abellon et al., 2014; Casella et al., 2010) by way of reducing response cost.

Finally, *delay* to a reinforcer can also reduce its value—more immediate reinforcers are often to preferred over those that are delayed (e.g., Mazur, 1987; Rachlin et al., 1976; Rasmussen et al., 2010). The behavioral mechanism of delay discounting (DD) is a relevant here. DD refers to a decrease in the value of an outcome as delay to its receipt increases (Ainslie, 1974, 1975). This process has been well characterized in nonhumans (see review by LeComte & Rasmussen, 2024) as well as humans, and is studied largely as a behavioral characteristic of those with substance use disorders (e.g., see Amlung et al., 2016b, 2016a, and Mackillop et al., 2011, for reviews) and obesity (see Amlung, Petker et al., 2016b, 2016a, and Rasmussen et al., 2024, for reviews).

Although this is not an exhaustive list of qualities that affect behavioral choice to various sources of reinforcement, they are well-established trends in the literature. It is noteworthy, however, to underscore the importance of alternative reinforcement inherent to each of these properties. At any point in time, there are many possible reinforcers that are competing for behavioral allocation. Often in research and practice, one behavior-reinforcer relation is chosen as a target, and other behavior-reinforcer relations are collectively referred to as alternative reinforcement, though in many laboratory studies a single alternative is selected for study to represent the aggregate of alternatives (e.g., Fontes et al., 2018; Hursh, 1984).

In reality, however, the properties of a target behavior-reinforcer relation and its alternatives are complex and probabilistic. For instance, if a university student in the intermountain west has the choice to attend class (a target behavior), the reinforcers of attending class (e.g., getting course-related information to obtain a passing grade, the inherent rewarding properties of the course topic, the wittiness of the instructor, friendly classmates) are part of the reinforcing relation. Inherent to this target behavior is the response cost (effort and delay) of getting to class (e.g., walking a few blocks to campus). However, the qualities of alternative behavior-reinforcer relations exist (e.g., sleeping in, streaming a favorite show, meeting friends for coffee, or going to work) and compete with this target. A snowstorm, a condition that creates greater response cost, time, and risk to get to campus, may combine with the practice of the professor who does not take attendance in a class (a potential absence of reinforcement for attendance). This may result in a student opting to sleep in on a cold winter’s day, one of the alternative behavior-reinforcer relations. However, a clear day combined with a scheduled exam worth many points towards a grade makes it much more likely that the student will attend class. Therefore, the target behavior is not just about the complex sum of properties of reinforcers and response costs inherent to maintaining class attendance, but also the net potency and response costs of accessing alternative reinforcers.

One well-documented effect of the introduction of alternative reinforcement is the reallocation of behavior to the reinforcers that are available, which often reduces allocation to the target response-reinforcer relation. This is especially the case if the available alternative reinforcers are potent enough to compete with the target. Nonhuman experiments with drug self-administration are excellent examples. The behaviors of drug seeking and drug self-administration are maintained by the reinforcing properties of the drug itself, accessibility (often effort-based) of the drug, but also the accessibility of nondrug reinforcers (see Bigelow et al., 1998). For example, in a study by Carroll et al. (1991), the behavior of monkeys was allocated to two lip-contact sippers in which liquid delivery occurred under concurrently available fixed ratio (FR) schedules that varied from 4 to 128 responses. Contact with one sipper delivered the drug phencyclidine (PCP) and contact with the other sipper delivered a competing alternative reinforcer—water, in some instances, or saccharin in others. PCP was most likely to be self-administered when it was available at a low price, such as FR 4, in which four responses were required for drug delivery. However, when price systematically increased to up to 128 responses, its consumption decreased. But this also depended on the price of the alternative nondrug reinforcer. When saccharin-flavored water competed for behavioral allocation at a lower FR, PCP consumption decreased even more. When water, a less potent nondrug alternative reinforcer, was available, water competed less with PCP for behavioral allocation, especially when the price of water was high (e.g., FR 128). Therefore, drug consumption depended highly on the accessibility of the drug (by way of response cost), and the accessibility and quality of competing reinforcers. A number of studies with different drugs and alternative nondrug reinforcers support these trends (e.g., Carroll, 1985; Carroll et al., 1989; Carroll & Lac, 1993; Cosgrove & Carroll, 2003; Ginsburg & Lamb, 2018; Khoddam et al., 2018; Nall et al., 2018; Quick et al., 2011).

The reduction in a target behavior when competing alternative reinforcers are introduced has also been shown in applied research with humans with substance use disorders (SUDs). For instance, generally low levels of alternative reinforcement, measured by endorsement of items on the Pleasant Events Schedule (MacPhillamy & Lewinsohn, 1974, 1982), are associated with marijuana use in adolescents (Khoddam et al., 2018). In addition, contingency management (CM) is an effective type of behavioral therapy that capitalizes on alternative reinforcement as a mechanism of reducing drug use. With CM, the target response of drug abstinence increases when abstinence is reinforced with nondrug reinforcement (see reviews by Davis et al., 2016; Higgins et al., 2019; Petry et al., 2017; and meta-analysis by Bolívar et al., 2021). Vouchers that are exchangeable for preferred goods and services are contingently delivered for drug-abstinent tests (e.g., urine test or a breath carbon monoxide test). The vouchers serve as reinforcers, but also give greater access to a broad range of preferred nondrug reinforcers that compete with drug seeking and self-administration of the drug. Often, the value of the voucher increases with each drug-abstinent response, such that the response cost for drug use (i.e., nonabstinence) increases systematically. The use of CM to reduce substance use has been highly effective at reducing consumption of a wide range of drugs including nicotine (see Secades-Villa et al., 2020, for review), cocaine (see Farronato et al., 2013; Schierenberg et al., 2012), marijuana (e.g., Carroll et al., 2006; Stanger et al., 2009), alcohol (e.g., Barnett et al., 2017; Petry et al., 2000), and opioids (see reviews by Bolívar et al., 2021; Higgins et al., 2019; Proctor, 2022).

## Reinforcer Diversity

The term “reinforcer diversity*”* might be a useful one. It is a conceptual, though measurable variable that refers to the *number and quality* of reinforcers that are typically contacted in a given period of time for an individual. Thus, reinforcer diversity is an extension of alternative reinforcement. Although alternative reinforcement may refer to either the aggregate of all possible other reinforcers or a single nontarget behavior-reinforcer relation selected from the aggregate (e.g., Ginsburg & Lamb, 2018; Khoddam et al., 2018; Nall et al., 2018), our conceptualization of reinforcer diversity (1) includes the target behavior-reinforcer relation and (2) also specifies the total number and quality of other (alternative) reinforcers contacted in a given time period. It is conceptual in that a person could likely report about their own reinforcer diversity within a duration of time and make a loose estimate about whether they are different than usual (e.g., “I have been so busy with work the last month that I have not had time for my family, friends, or even the gym.”). Health care professionals ask about reinforcer diversity with their clients, for example, when asking about depressive symptoms (especially anhedonia) and whether a patient has noticed that they are engaged in fewer of their normally enjoyed activities (i.e., contacting fewer of the reinforcers) than is typical. Reinforcer diversity could also be an empirical term, in that quantifying the number and quality of reinforcers that a person interacts with in a period of time is measurable.

Examples of number and quality may illustrate the use of this term. When reinforcement diversity is too low, this means that quantity and quality of reinforcement is low. For example, consider a typical 24-hr period for an Olympic athlete in training. The athlete may typically spend 10–12 hr of their day training for their sport (also subjectively highly valued) and 2 hr eating (subjectively moderately valued, though important to training), and 9 hr sleeping (highly valued, especially after training); smaller amounts of time are spent in transit or in smaller, though less valued tasks, such as tidying the house. Because of the athlete’s cumbersome training schedule, which requires a great deal of physical activity and eating to maintain energy, they have time for only two, perhaps three, valued reinforcers in a day, and two of them (eating and sleeping) are necessary for high performance of the third (training). The athlete also forgoes time for other sources of subjectively valued reinforcement, such as engaging in hobbies or recreation, school, or spending time with family or friends.

Another example of low reinforcement diversity may be a person in the throes of a severe substance use disorder (SUD), in which substance seeking and substance use may be highly probabilistic behaviors (14–16 hr/day) maintained by one reinforcer—the substance (subjectively highly valued). Other subjectively valued reinforcers, such as employment, schooling, connective social interactions, self-care (e.g., sleep and healthy eating), and hobbies may be contacted less. In both situations, overconsumption of one behavior-reinforcer relation occurs at the expense of other valued reinforcers. (As a side note, society values the athlete’s behavioral pattern over the person with the SUD, though both are forms of overconsumption of one reinforcer.)

On the other hand, a person may have higher reinforcer diversity. In this situation, an individual may interact with a large number of different, but highly valued reinforcers within a time period. For instance, a person may have full-time employment at a job they value (8 hr), but they also have time for visiting with friends or family (1–2 hr), engaging in physical activity they find pleasurable (1 hr), and developing a hobby (1–2 hr) they enjoy. They also read a book for pleasure (1 hr) before getting a full night’s sleep (8 hr), all of which they enjoy. All six of these reinforcers are subjectively highly valued by the individual.

A behavioral measure of reinforcer diversity would require following a person in real time during the day (and arguably at night), and recording the number of reinforcers with which they make contact, how much time they spend engaging in each specific behavior-reinforcer relation, while querying on their respective values. In practice, this would be difficult and therefore impractical. A more realistic option might be a self-report behavioral checklist. Although not as scientifically robust, a checklist has the benefits of ease and quickness of measurement. To date, we know of only one such measure—the Pleasant Events Schedule (MacPhillamy & Lewinsohn, 1974, 1982), which is a self-report behavioral inventory with hundreds of items that are quite specific (e.g., “playing with pets” or “going to a play”). Participants rate the frequency and subjective likeability of these items as 0, 1, or 2 for both properties. This scale has strong reliability across 3 months, as well as predictive and construct validity and has been used as a measure of alternative reinforcement density in patients who are at risk for mental health problems, such as patients with depression or Alzheimers disease (e.g., Sweeney et al., 1982; Logsdon et al., 1997). There is also a modified version for elderly individuals with fewer items (Rider et al., 2016; Scogin et al., 2016; Teri & Lewinsohn, 1982). It is important to note, though, that this measure was originally developed at a time before many technological advances (e.g., social media, gaming, smart phones, computers, and the internet) have become an integral part of most people’s lives; therefore, these technological items are missing. In addition, completing a measure with hundreds of items twice (i.e., once for frequency and once for likability) may be time-consuming and burdensome for participants.

It is our contention, then, that reinforcer diversity have some practical utility. We believe its use may be especially helpful in the context of challenges to health and mental health, especially in situations when access to varying sources of reinforcement may change abruptly or gradually. One such situation is during a global pandemic in which quarantines are implemented.

## COVID-19 Quarantine, Behavioral Economics, and Changes in Reinforcer Diversity

COVID-19 quarantine policies included stay-at-home orders or isolation periods from typical out-of-home activities. These adaptations, though supported by public health research, restricted access to in-person work-related activities for many adults outside of the home environment. Moreover, students who normally attended school outside of the home instead learned remotely (i.e., online), which restricted group learning, social interaction, and extracurricular activities. Likewise, access to public activities (e.g., community businesses, services in the community, sports and recreation, church) and entertainment (e.g., movie theaters, bars, concerts) was also limited. Although the COVID-19 quarantine duration was temporary and varied greatly depending on geographic location, some COVID-19 restrictions and guidelines remained in place after quarantine to continue limiting viral spread until a vaccine was widely available (e.g., 2021 in the United States), though guidelines remained in place for longer durations for select areas with higher populations. For example, telecommuting, social distancing, and curbside pickup remained in place for some areas. Therefore, though restrictions eased after the quarantine, it is important to note that there were still a number of manners in which reinforcement remained restricted, depending on geographic location.

From a behavioral economic standpoint, a quarantine would predict a number of changes on reinforcer availability and behavior for members of a community or culture. First, a quarantine would predict an increase in the consumption of more accessible reinforcers (i.e., those in the home) and a decrease in the consumption of less accessible reinforcers, especially those restricted by policy. Second, these two trends would likely lead to a net reduction in reinforcer diversity during the quarantine. Third, a quarantine-induced net reduction in reinforcer diversity would likely result in health and mental health consequences for some members of the affected population. We address these first two predictions in this section. A number of descriptive studies have characterized how the consumption of specific, more accessible reinforcers increased during the COVID-19 quarantine. These key reinforcers include social media, sleep, substances, and food.

### Social Media

As quarantine lockdown and social distancing measures were implemented worldwide, traditional face-to-face social interactions appeared to be replaced by digital forms of communication (Brailovskaia et al, 2021; Delogu et al., 2025). Places for physical gatherings (e.g., workplaces, schools, religious centers, social venues) were closed or limited, leading to a greater reliance on digital communication. These included video conference platforms (e.g., Zoom), messaging applications, and digital applications, such as social media, for communication and dissemination of news (Saud et al., 2020). Individuals also found creative ways to maintain social bonds, such as virtual game nights, online classes, and Zoom karaoke (Heikkinen from Singa, 2023). Indeed, the annual convention for the Association for Behavior Analysis International (ABAI) was held via a virtual conference platform in 2020 in lieu of the typical in-person convention in a large city.

Research focusing on changes in social media use before and after the COVID-19 pandemic highlight the descriptive nature of how quarantine may have affected social connectiveness (i.e., social reinforcement) and mental health. A number of studies show that social media use increased dramatically in adults and children during the quarantine period (Brailovskaia et al, 2021; Delogu et al., 2025; Saud et al., 2020; Tsao et al., 2021). These studies suggest the specific functions of this increase were related to seeking social support, seeking information, and reduction of fear. We wish to also point out, however, that social media was more highly accessible because people were home, and therefore, there was less competition from other reinforcers related to school and work.

### Sleep

Sleep is complex in terms of its categorical fit into behavioral nomenclature. Sleep is biologically essential to health, in terms of having restorative function, building immunity, enhancing toxin removal, and consolidating learning processes (Irwin, 2015). Similar to other biologically important behaviors (e.g., eating), sleep likely has a reinforcing function. For instance, both humans and nonhumans, when sleep deprived, will engage in sleep seeking (e.g., searching for safe, quiet areas for rest) and sleep preparatory behaviors, such as closing one’s eyes (Axelsson et al., 2020). Sleep may also have a negatively reinforcing function, in that one may escape aversive life circumstances by sleeping more frequently, such as what may be observed during depressive episodes or a traumatic event (American Psychiatric Association, 2013). In addition, sleep is probabilistic; its occurrence can change when there are competing reinforcers (such as a cram session for a final exam; Huang et al., 2016; Siagian, 2022). Yet, it does not pass the dead man’s test (Lindsley, 1991, though see Critchfield, 2016), in terms of being categorized as actual behavior. We would therefore argue that sleep is best categorized as a biologically important reinforcer, though may also be considered a behavior, as its probability may also be reinforced (see unpublished dissertation by Ice, 2023; McLay & Lang, 2022).

Based on this assertion, a quarantine that restricts out-of-home alternative reinforcers would predict that sleep would increase. This is indeed what was generally observed during the COVID-19 quarantine with young adults and adolescents (Socarras et al., 2021). Adolescents in the United States reported longer sleep durations and delayed waketimes (“sleeping in”) during the quarantine compared to pre-pandemic times (Becker et al., 2021). This was also the case with teens in Iran, who reported >12 hr of sleep per day during the early part of the pandemic (Ranjbar et al., 2021). In some European countries, this was also documented. Young adults in Spain (Sañudo et al., 2020) and university students in Italy tended to go to bed later and sleep in later (Marelli et al., 2021). These patterns across studies show longer sleep durations.

### Alcohol and Substances

There is evidence that the COVID-19 quarantine was also associated with increased alcohol and substance consumption. Studies show that alcohol consumption increased during the COVID-19 quarantine compared to pre-pandemic times (Almandoz et al., 2021; Castaldelli-Maia et al., 2021; Grossman et al., 2020). Although in many areas in the United States, liquor stores, bars, and restaurants were closed during quarantine, retail alcohol sales increased by 34%–54% in the early stages of the pandemic (Moskatel & Slusky, 2023). Online alcohol sales also increased two- to three-fold at the beginning of the quarantine (Furni, 2020). To limit the frequency of purchase, some consumers ordered alcohol in bulk, which enhanced the amount of alcohol available in households (Nielsen, 2020; Pollard et al., 2020). In addition, many states altered policies on carry-out purchases of alcohol to assist with the financial impact on restaurant businesses. For instance, the Idaho State Police Bureau of Alcohol Beverage Control allowed the sale of curbside mixed drinks in businesses that sold alcoholic beverages, with a few provisions (Rives, 2020). It is noteworthy that during the pandemic those who worked from home or lost employment were most likely to increase alcohol consumption; those who continued to work in-person during the quarantine did not experience such an increase (Szajnoga et al., 2020). In addition, alcohol use during the quarantine was reported to reduce aversive symptoms associated with COVID-induced distress (e.g., stress, anxiety, and depression; Capasso et al., 2021), a type of negative reinforcement.

This negative reinforcement function may also explain why some individuals also reported engaging in substances other than alcohol over the COVID quarantine. Marijuana use increased by 20% among emerging adult marijuana users who self-isolated during quarantine (Bartel et al., 2020). Indeed, people who use illicit substances reported a 26% consumption increase during quarantine compared to pre-quarantine (Chacon et al., 2021). In addition, there was a 16% increase in drug use for individuals who had preexisting drug dependence before the pandemic. These trends will be explicated in more detail later in this article when substance trends and their health implications are described.

### Food

Research has also shown that dietary behaviors changed during the COVID-19 pandemic. There were several major trends reported. First, fruit consumption significantly decreased during quarantine compared to pre-pandemic times (Jafri et al., 2021). The quarantine lockdown disrupted fresh food supplies by slowing the harvest of fruits and vegetables and their transport. Some businesses that sold healthier, though perishable, foods were also closed or limited (Jafri et al., 2021). Second, consumption of nonperishable foods (e.g., canned and processed foods) increased (Jafri et al., 2021). In other words, many individuals purchased and stocked processed and canned foods with added sugars and preservatives, rather than fresh alternatives. Third, there was greater consumption of foods that were processed and high in sugar, including “comfort food”, which contains high fat, sugar, salt, and carbohydrate content (Jafri et al, 2021; see review by Jehi et al., 2023). This trend was also linked to eating during subjective feelings of emotional distress, loneliness, and depression (Ederer et al., 2023; Pellegrini et al., 2020; Salazar-Fernández et al., 2021). One study from an obesity unit in Italy found that “comfort eating” during quarantine was especially high in patients with obesity (Pellegrini et al., 2020).

Third, food insecurity, or reports of inconsistent access to healthy food was predicted by experts before the quarantine took place, because previous quarantines tend to predict higher food insecurity, especially with those who are already struggling with financial challenges (Chu et al., 2020). Indeed, this trend was supported in college students, in which food insecurity increased by 54% (Sidebottom et al., 2021; see review by Jehi et al., 2023). Noncollege adults in the United States were also more likely to be food insecure during quarantine compared to pre-quarantine (Bin Zarah et al., 2020). Food insecurity is linked to consumption of ultra-processed food, which is more affordable than fresh produce and lower-fat meats, and this trend was also found with food-insecure individuals during the COVID-19 quarantine (see review by Jehi et al., 2023). These patterns followed for people in other parts of the world as well, including Jordan (Elsahoryi et al., 2020), the Philippines (O’Meara et al., 2024), and the United Arab Emirates (AlBooshi et al., 2023).

### Some Additional Data that Support These Trends

Descriptive research trends about quarantine-related changes in behavior reveal that social media usage, sleep, and consumption of alcohol, substances, and processed food increased over the COVID-19 quarantine. We conducted a retrospective within-subjects study with 51 undergraduate students at Idaho State University (Pocatello, ID) about a year after the COVID-19 quarantine (see Table 1 for demographic characteristics from this sample). Data from this study also support these trends. In particular, we investigated the extent to which the COVID-19 quarantine may have restricted access to, and changed the self-reported consumption of some common reinforcers.

**Table 1:** Participant Demographics

	Total (*n* = 51) Mean (SEM)
Sex^a^ (%Female)	*n* = 51 (66.7%)
Ethnicity^a^ (%White)	*n* = 39 (75%)
Hispanic or Latino	*n* = 9 (17%)
American Indian or Alaskan Native	*n* = 1 (2%)
Black or African American	*n* = 1 (2%)
Other	*n* = 1 (2%)
Mean Age	20.04 (0.65)
Age^a^ (< 21 yrs.)	60.80%
%Income > 70,000^a^	35.3%
Mean BMI (kg/m^2^)	26.74 (0.86)

To approximate a measure of reinforcer diversity, we asked each participant, using a visual analog scale presented via computer, to rate the amount of time they typically spent engaging in a number of different activities (i.e., reinforcers), as well as the subjective value of each reinforcer using a Likert scale (0 = no time; no value and 5 = highest amount of time; highest value)—see Appendix for measure. They made these ratings at three time points: before the quarantine, during the quarantine, and after the quarantine.

We analyzed trends according to whether these reinforcers were restricted or accessible. Restricted reinforcers appeared in definitions of nonessential businesses in the State of Idaho (Little, 2020), which were mandated to close during quarantine. Accessible reinforcers referred to those found in the home or deemed accessible as an essential business, which under State of Idaho definition was allowed to remain open during quarantine (Little, 2020). In addition, we asked participants how many meals per day they ate and how often they used alcohol and illicit substances per week. These reinforcers are listed below:

- Restricted Reinforcers:
  - Extracurricular School Activities
  - In person-socialization with Friends
  - Public Entertainment
  - Travel
  - Church Activities
  - Employment
- Accessible Reinforcers:
  - Social media usage
  - Sleep
  - Meals
  - Alcohol
  - Illicit drugs

**Fig. 1:**
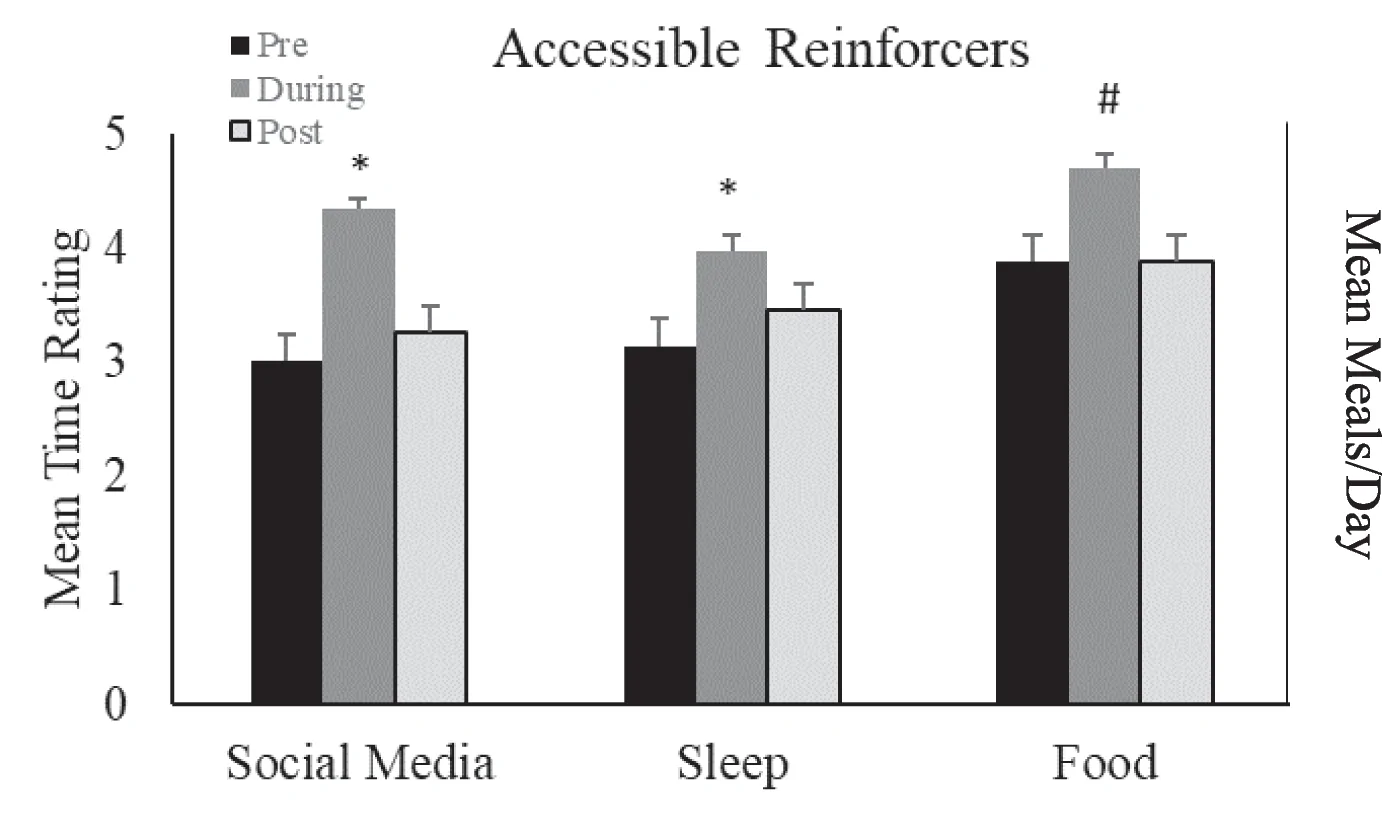
Shows mean time ratings (Likert scale) for social media and sleep as a function of COVID-19 quarantine time period: pre (black), during (dark gray), and post (light gray). Mean meals per day (Food) is shown as a second y-axis. Error bars = 1 SEM. *p < 0.001; #p < 0.05. Social media* (*F(49)= 20.96, *p*< 0.001, η^2^=.46), sleep (F(49)=26.29, *p* < 0.001, η^2^=.52), and food (F(49)= 6.48, *p*= 0.003)

Time and value data for each reinforcer were significantly correlated (*r* (range): 0.45–0.85) and similar in terms of effects, so to simplify, time only is shown as the main dependent variable. Figure 1 shows participants’ self-reported rating of time on social media (left), sleep (middle), and food (meals per day, right y-axis) significantly increased over the COVID quarantine period compared to baseline. A return to baseline for each of these reinforcers was also observed after the quarantine ended.

Figure 1 shows mean time ratings (Likert scale) for social media and sleep as a function of COVID-19 quarantine time period: pre- (black), during (dark gray), and post- (light gray). Mean meals per day (Food) is shown as a second y-axis. Error bars = 1 SEM. *p < 0.001; #*p* < 0.05. Social media (*F*(49) = 20.96, *p* < 0.001, η^2^ = .46), sleep (*F*(49) = 26.29, *p* < 0.001, η^2^ = .52), and food (*F*(49) = 6.48, *p* = 0.003)

**Fig. 2:**
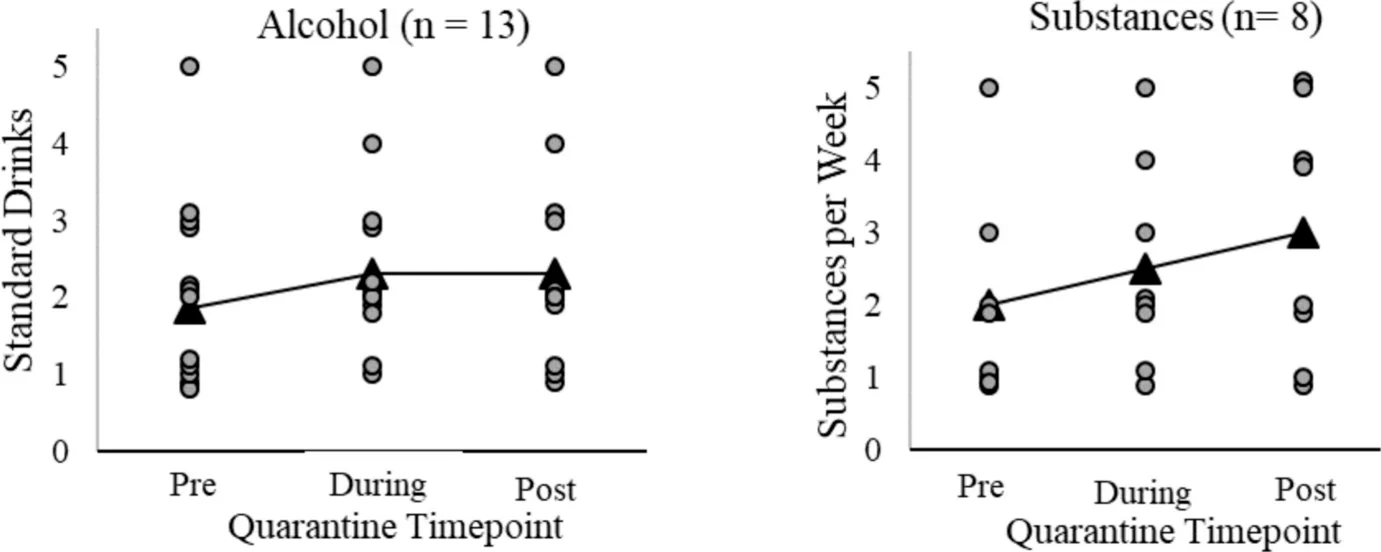
Standard Drinks (left) and Substance Use per Week (right) as a Function of Quarantine Time Point for Alcohol. *Note.* Only 13 and 8 students reported drinking and substance use, respectively, in the data set

Data on alcohol (*n* = 13) and substances (*n* = 8) are presented in Figure 2, though they are limited by the lower number of individuals in the study who use these substances; analyses are therefore statistically underpowered. Therefore, we report these as individual data, as opposed to means. The left panel of Figure 2 shows the self-reported number of drinks per week for the 13 participants who reported consuming alcohol as a function of quarantine time point. Means for each timepoint are represented as triangles. The number of drinks slightly rose during quarantine and remained similar post-quarantine. The right panel of Figure 2 shows the number of times per week individuals consumed illicit substances across quarantine time point for the eight individuals who self-reported using illicit substances. Substances per week rose across timepoint.

These data show that the more accessible behavior-reinforcer relations of social media, sleep, and food increased significantly over quarantine, then decreased to near-baseline levels after the quarantine. Some limited data with participants who reported consuming alcohol and substances show that consumption increased slightly during quarantine, but did not return to baseline after quarantine. All of these trends, however, are consistent with elevated consumption of these reinforcers during quarantine (e.g., Almandoz et al., 2021; Brailovskaia et al, 2021; Castaldelli-Maia et al., 2021; Chacon et al., 2021; Delogu et al., 2025; Jehi et al., 2023; Socarras et al., 2021).

## Restricted Reinforcers

In addition to increases in reinforcers that were accessible, we present some data on reinforcers that were restricted during quarantine. Restricted reinforcers included those described in the local list of state-based nonessential businesses that were required to close during quarantine. These include public entertainment such as movies, concerts, etc., churches, and activities that were specifically limited during quarantine, such as travel. In addition, because universities were closed (i.e., transitioned to remote learning), extracurricular activities, such as clubs and sports, were restricted (Little, 2020).

**Fig. 3.**
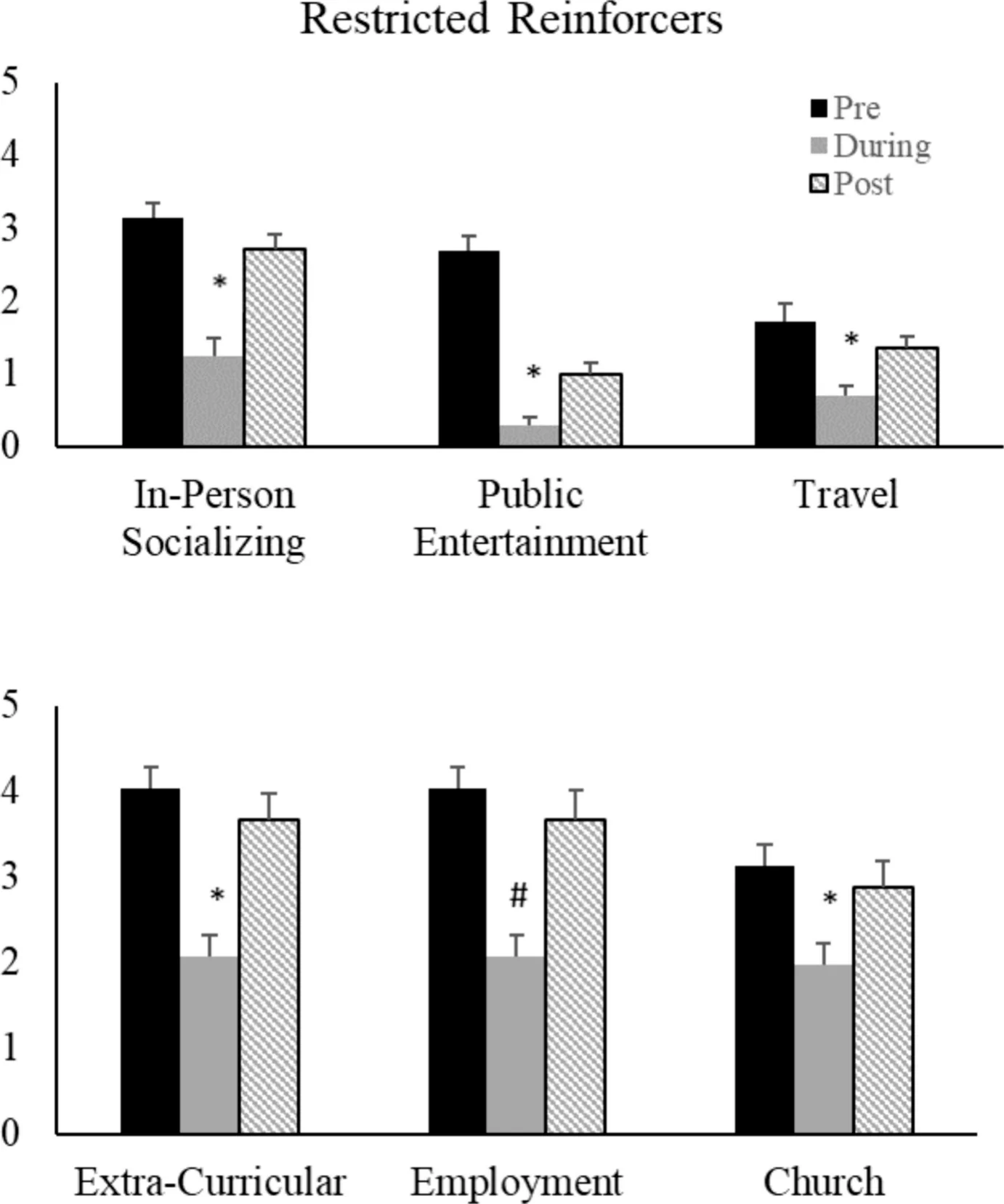
Mean Self-Rated Time for More Restricted Reinforcers as a Function of Pre-, During, and Post-Quarantine. *Note.* Error bars = 1 SEM. *p < 0.001; #*p* < 0.05. In-person socialization (*F*(49) = 31.61, *p* < 0.001, *η*^*2*^ = .563), pubic entertainment public entertainment (*F*(49)= 53.78, *p* < 0.001, *η*^*2*^ = .687), travel (*F*(49) = 13.79, *p* < 0.001, *η*^*2*^ = .36), extracurricular school activities (*F*(49) = 28.4 , *p* < 0.001, *η*^*2*^ = 537), employment (*n* = 46; *F*(44) = 4.78, *p* = .017, *η*^*2*^ = .17), and church (*n* = 32; *F*(30) = 11.83,* p* < 0.001, *η*^*2*^ = .44)

Figure 3 shows mean time ratings for six restricted reinforcers as a function of quarantine timepoint. The top panel shows in-person socialization, public entertainment, and travel; the bottom panel shows extracurricular school activities, employment, and church. The figures show that mean time ratings were significantly reduced during quarantine compared to before (pre-) and after (post-) the quarantine (see figure caption for full statistics). This was the case across all six reinforcers.

Thus, the results show that during quarantine, time allocated to reinforcers that were restricted by quarantine policies was reduced, which likely allowed for the higher allocation of time to the more accessible reinforcers. One question that arises from these two trends is the extent to which quarantine would net an overall change in reinforcer diversity during the quarantine. We would predict a net reduction in reinforcer diversity during quarantine compared to pre- and post-quarantine timepoints. We attempted to answer this question by summing the number of reinforcers from the list that received both *time and value* ratings above 3 for each individual and statistically comparing them across the three timepoints.

**Fig. 4:**
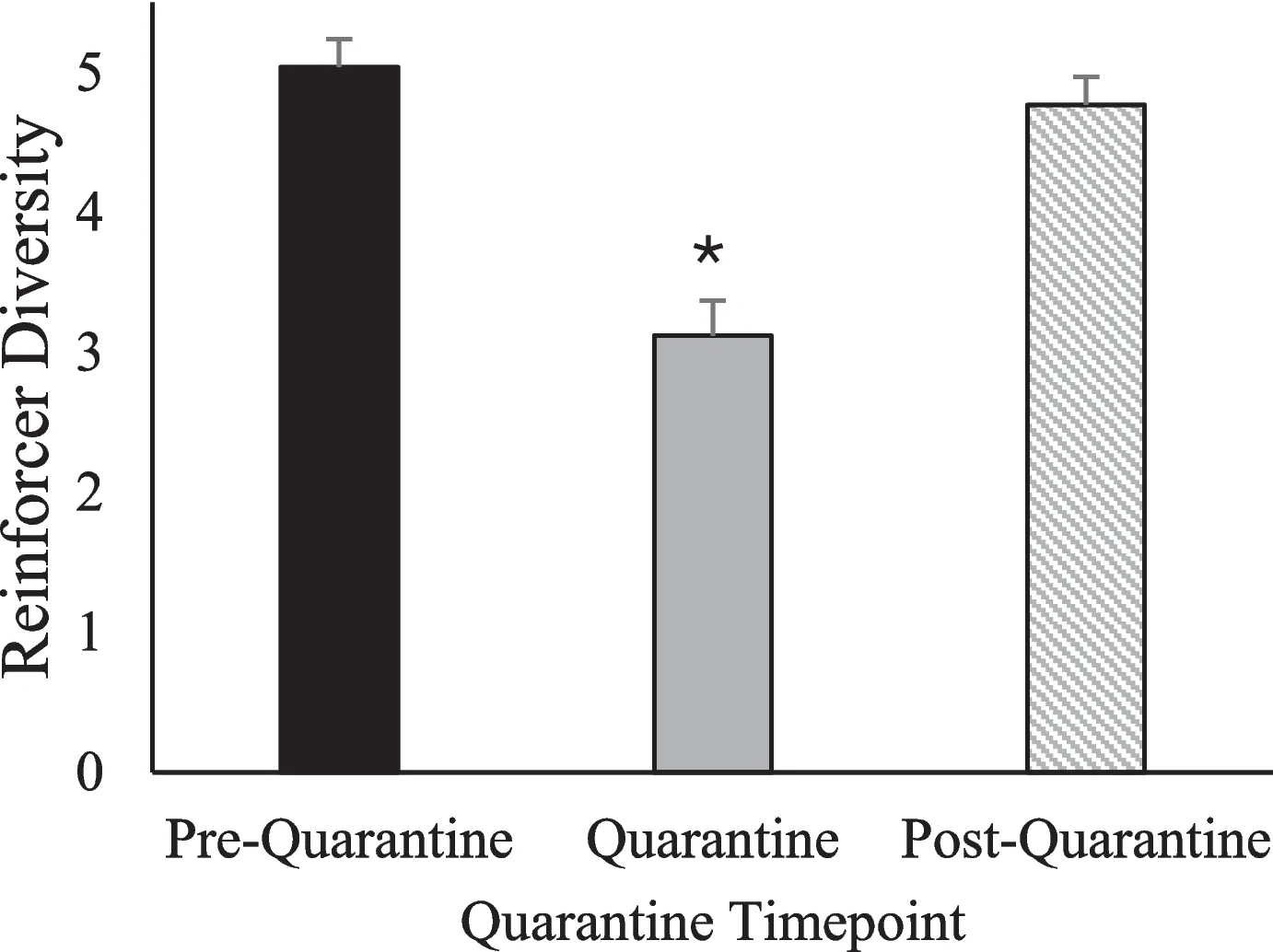
Reinforcer Diversity (Number of Reinforcers Rated >3 for Time and Value) Compared across Three Timepoints: Pre-, During, and Post-Quarantine. *Note.* Reinforcer diversity decreased significantly during quarantine, but recovered post-quarantine *(*F*(49) = 28.4, *p* < .001, *η*^*2*^ = _._57) Error bars = 1 SEM

Figure 4 shows reinforcer diversity across quarantine timepoint. Before quarantine, individuals rated, on average, five reinforcers as valued and time-worthy. During quarantine, this number decreased significantly to just above 3, though returned to baseline levels after quarantine. These data support that reinforcer diversity was reduced by 40% during the quarantine, though recovered afterward.

These within-subject data show, then, that reinforcer diversity decreased during quarantine. Moreover, this net effect was likely due to higher self-reported consumption of accessible reinforcers and lower consumption of restricted reinforcers during quarantine. No study to date offers this behavioral economic account of these changes in reinforcement consumption.

We feel it is important to note the limitations of these data. First, as mentioned, the nature of the study was retrospective and based on self-report. Data were collected one year after the COVID-19 quarantine period for these participants ended. In an ideal situation, behavioral data should be collected in real time as it happens, so that the most accurate characterization is possible. However, given the abrupt nature of the pandemic, it would have been impossible to design, get approval from an institutional review board, and collect data with participants, especially as the quarantine unfolded. Though limited, retrospective self-report studies have a place in conducting research when collecting behavioral real-time data is not possible. For example, predictive information can be gleaned from individuals when they are given a calendar and asked to recall their patterns of drinking and indeed can be used to accurately predict behaviors (Martin‐Willett et al., 2020).

A second limitation is that the theoretical characterization of the data assumes that one reinforcer is *substitutable* for another reinforcer. A reinforcer, however, may have interactive properties with other reinforcers. For instance, reinforcer consumption that is *complementary* depends on, and occurs in the same direction as, consumption of another reinforcer (Hursh, 1980, 2014). Consider, for example, the behavior-reinforcer relations of exercise and social interaction for an individual. That is, a person may only exercise in social settings with friends present, such as group fitness classes at a gym. When gyms are closed and exercise consumption decreases, so too will social interaction for that individual. On the other hand, if no one is available to exercise with that individual because of a quarantine, that person may be much less likely to attend fitness classes at a gym that happens to be open. Therefore, consumption of one reinforcer is tied heavily to the consumption of the other. Reinforcer consumption may also be *independent* of other reinforcers, too (Hursh, 1980, 2014). It is therefore important to acknowledge that some reinforcers may, or may not, interact with consumption of other reinforcers in a variety of ways.

A third limitation to these data concerns their generalizability. The data reported here are based on college students, who were mostly female and white. Participants also resided in a smaller city in rural Idaho (population around 60,000). Therefore, the extent to which these data generalize to larger cities with longer quarantine periods and more diversity, such as New York City or Chicago, is unclear.

### Quarantine-Induced Decreases in Reinforcer Diversity and Health Effects

The research trends and data from our study suggest that the quarantine period of the COVID-19 pandemic caused a net reduction of reinforcer diversity compared to pre- and post- quarantine timepoints. One question that remains is the extent to which this quarantine-induced reduction in reinforcer diversity may have been linked to health and mental health effects during that time period. Although no data were collected on this question within our study, trends in the extant literature may provide valuable information.

#### **Anxiety and Depression**

A number of studies with both humans and nonhumans describe the pivotal role that reductions in reinforcement play in the development of depressive symptoms (e.g., Carvalho et al., 2011; Huston et al., 2013). With humans, anxiety is often co-morbid with depressive symptoms and there is evidence that one characteristic of anxiety—difficulties in extinguishing responses to fear cues—is also related to neuromechanisms involved in depression (Dibbets et al., 2015; Tronson et al., 2008). Therefore, quarantine-related reductions in reinforcement may have combined with fear cues associated with a rapidly spreading disease to increase depressive and anxiety symptoms. Indeed, even before the COVID-19 quarantine, a few studies on previous epidemics were used to draw speculations about the possible effects that the COVID-19 quarantine might have on health and mental health outcomes in children. For example, children and adults who were isolated during the Severe Acute Respiratory Syndrome (SARS) epidemic in Toronto in the early 2000s showed increased anxiety and depressive symptoms during a quarantine period (Cava et al., 2005; DiGiovanni et al., 2004).

Studies on the occurrence of anxiety and depressive symptoms during the COVID-19 quarantine indeed reveal increases in these mental health outcomes in the United States. In particular, there were increases in COVID-19 related fears (e.g., fear of infection or infecting others), but also various types of reinforcement loss, such as emotional loss (e.g., loss of loved ones, loss of relationships) and loss of structure (e.g., losing routines or jobs); these contributed to an increased risk of anxious and depressive symptoms (Guo et al., 2020; Samji et al., 2022). There was also a higher incidence of post-traumatic stress and insomnia reported during quarantine (Guo et al., 2020). These conditions may also increase the risk of major depression, suicidal ideation, as well as suicide attempts. In addition, some segments of the population were especially affected mental health-wise by the quarantine. For instance, adolescents who were diagnosed with ADHD before COVID quarantine reported greater levels of stress, anxiety, and depression compared to before the quarantine (Becker et al., 2021).

Thus, the general trends in mental health research point to higher incidences of depressive and anxious symptoms during the COVID-19 quarantine. It would be important to determine the extent to which these mental health concerns were also tied to the overconsumption of the key reinforcers described earlier in this article (i.e., social media, sleep, substances, and food). These are described next.

#### **Social Media Use and Psychological Symptoms**

Pandemic aside, research shows a correlation between higher social media use and an increased risk of mental health problems, such as distress, depression, cyberbullying, self-harming behaviors, and suicidality, especially with adolescents (see reviews by Khalaf et al., 2023; Schønning et al., 2020). However, online communication and social media also may have positive effects, including enhancement of social connectivity, especially if the communication is from high-quality relationships, such as close friends or family. Therefore, two sets of predictions might be gleaned from increased social media use during the quarantine: one may be more harmful to mental health and one may be more protective.

As mentioned previously, social media use increased substantially during the COVID-19 quarantine compared to pre-quarantine timepoints (Brailovskaia et al., 2021; Delogu et al., 2025; Saud et al., 2020; Tsao et al., 2021). This increase, in connection to mental health endpoints, however, was nuanced. One review showed an association between social media use and feelings of distress over the quarantine (Marciano et al., 2022). Another study showed that, during quarantine, COVID-19-related anxiety was lowest in adolescents who checked their social media less frequently, but also had accurate information on the pandemic (Mousavi et al., 2023). Those with the greatest anxiety tended to use social media as a way to cope with aspects of the pandemic, such as fear of contracting the virus or domestic issues, such as family conflict (Cauberghe et al., 2021). Other studies, however, showed that social media use during quarantine played a critical role in maintaining connectedness to others and coping with loneliness (Cauberghe et al., 2021; Gabbiadini et al., 2020; Pancani et al., 2021; Salzano et al., 2021; see also review by Marciano et al., 2022). Therefore, increased social media use that enhanced greater quality of social connection appeared to have protective effects on mental health, whereas other types of social media use (e.g., excessive newsseeking or “doomscrolling”) were associated with greater amounts of anxiety (Buchanan et al., 2021; see George et al., 2024).

#### **Sleep and Psychological Challenges**

As mentioned, time spent sleeping generally increased over the quarantine (e.g., Becker et al., 2021; Socarras et al., 2021), and the most prevalent sleep pattern change was delayed sleeping over the pandemic (i.e., going to sleep later than usual and “sleeping in”; Becker et al., 2021; Marelli et al., 2021; Ranjbar et al., 2021; Sañudo et al., 2020). More sleep seemed to benefit children, who were better able to sync their sleep patterns to their natural circadian rhythms (Pilcher et al., 2022). However, more sleep may not necessarily have been beneficial for mental health for adults. Over the COVID-19 quarantine, adults who delayed sleep reported longer sleep durations, but worse sleep quality (Gupta et al., 2020).

Decreases in sleep quality were documented in many countries during the quarantine, including Jordon, Japan, China, the United States, and Italy (AlRasheed et al., 2021; Barrea et al., 2020; Fong et al., 2023; Fu, et al., 2020; Saadeh et al., 2021; Zhao et al., 2021). Poor sleep quality included delayed sleeping, but also sleep inefficiency, sleep disturbances, and poor sleep quality-induced daytime dysfunction. Poor sleep quality in college students and adolescents during the quarantine was associated with greater depressive symptoms (AlRasheed et al., 2021; Saadeh et al., 2021). Sleep disorders in adults and children increased over the pandemic, and were related to higher levels of anxiety and psychological distress (Casagrande et al., 2020; Fong et al., 2023).

#### **Substances and Substance Use Disorders**

Alcohol, marijuana, and illicit drug use increased during the pandemic, especially during the early stages of the quarantine (e.g., Almandoz et al., 2021; Castaldelli-Maia et al., 2021; Chacon, 2021; Grossman et al., 2020). Indeed, the shift to greater amounts of drinking led to problematic health trends for some people. For instance, binge drinking, which refers to four or more drinks for women and five or more drinks for men in a short period of time (CDC, 2024d), increased by 34% during the COVID-19 pandemic compared to pre-pandemic times (Grossman et al., 2020). Binge drinking is problematic because it can increase the risk of blackouts, accidents, overdose, and unsafe sexual behavior (NIAAA, 2025). Moreover, binge drinking can increase the risk of liver disease, as well as certain types of cancer, including esophageal, liver, and colorectal cancers (NIAAA, 2025).

Episodes of heavy drinking also increased by 41% during quarantine in women (RAND, 2020). Heavy drinking refers to 8 or more drinks per week for women or 15 or more drinks per week for men (CDC, 2024d). Heavy drinking carries risks similar to binge drinking, but chronic heavy drinking also increases the odds of heart disease, liver disease, stroke, alcohol use disorder, weakened immunity, memory problems, and relationship dysfunction (CDC, 2024d). Therefore, a continued pattern of heavy drinking increases the risks of health problems.

Although binge drinking and heavy drinking increased during the pandemic, perhaps the largest substance-related health problems during quarantine were documented in individuals who were already diagnosed with substance use disorders (SUDs), especially at the initial outbreak of COVID-19 (Avena et al., 2021; Hulsey et al., 2020). Indeed, SUDs are often comorbid with mental health struggles, such as depression, anxiety, and other challenges (see reviews by Lai et al., 2015, and Vorspan et al., 2015). Within the context of the COVID-19 quarantine, about 50% of individuals with SUDs also experienced symptoms of psychological disorders and reported using substances as ways to cope with symptoms (Chacon et al., 2021). This population also reported reductions in access to treatment and recovery services, including support groups, during the quarantine, which may have exacerbated mental health problems (Avena et al., 2021).

With individuals who use substances, higher risk of illicit drug overdose was another health risk associated with the quarantine. Consider the research on those with opioid use disorder. When comparing data from Medicare beneficiaries from September 2018 to February 2020 (as a pre-pandemic cohort) to a second cohort from September 2019–February 2021 (pandemic cohort), a higher percentage of individuals with opioid use disorder overdosed in the pandemic cohort compared to those in the pre-pandemic group (CDC, 2023). Indeed, the CDC (2021) estimated a record number of U.S. drug-related deaths in 2020.

In addition to drug overdose, there were other challenges identified during the quarantine period that increased health risks for those more vulnerable to SUDs (Mellis et al., 2020; Ornell et al., 2020). For instance, access to treatment services was hindered by the quarantine. Because hospitals were filled beyond capacity for treating individuals with severe COVID-19 symptoms, those with substance use disorders were deprioritized for emergency care. Although in-person treatment for SUDs was offered virtually, many who needed these services did not have regular access to a computer or the internet. In addition, harm-reducing approaches like naloxone and needle exchanges were also less accessible during quarantine (Hulsey et al., 2020; Mellis et al., 2020). These restrictions likely detrimentally affected health in this population during quarantine.

#### **Food and Weight Gain**

As mentioned previously, the COVID-19 quarantine resulted in greater consumption of processed food and lower consumption of fruits and vegetables (e.g., Ederer et al., 2023; Jafri et al., 2021; Jehi et al., 2023; Pellegrini et al., 2020; Salazar-Fernández et al., 2021). This resulted in weight gain for many. Trends in the United States, for example, showed that weight and BMI significantly increased during the pandemic compared to pre-pandemic times (Lange et al., 2021; Urzeala et al., 2021). On average, individuals in the United States gained 1.8 pounds per month during the quarantine period (Lin et al., 2021). Another study showed that people with moderate or severe obesity gained an average of 7.3 pounds in a span of 6 months, compared to healthy weight participants who only gained 2.7 pounds in the same span (Lange et al., 2021; Urzeala et al., 2021). This suggests that those already struggling with weight were at even greater risk for weight gain.

**Fig. 5:**
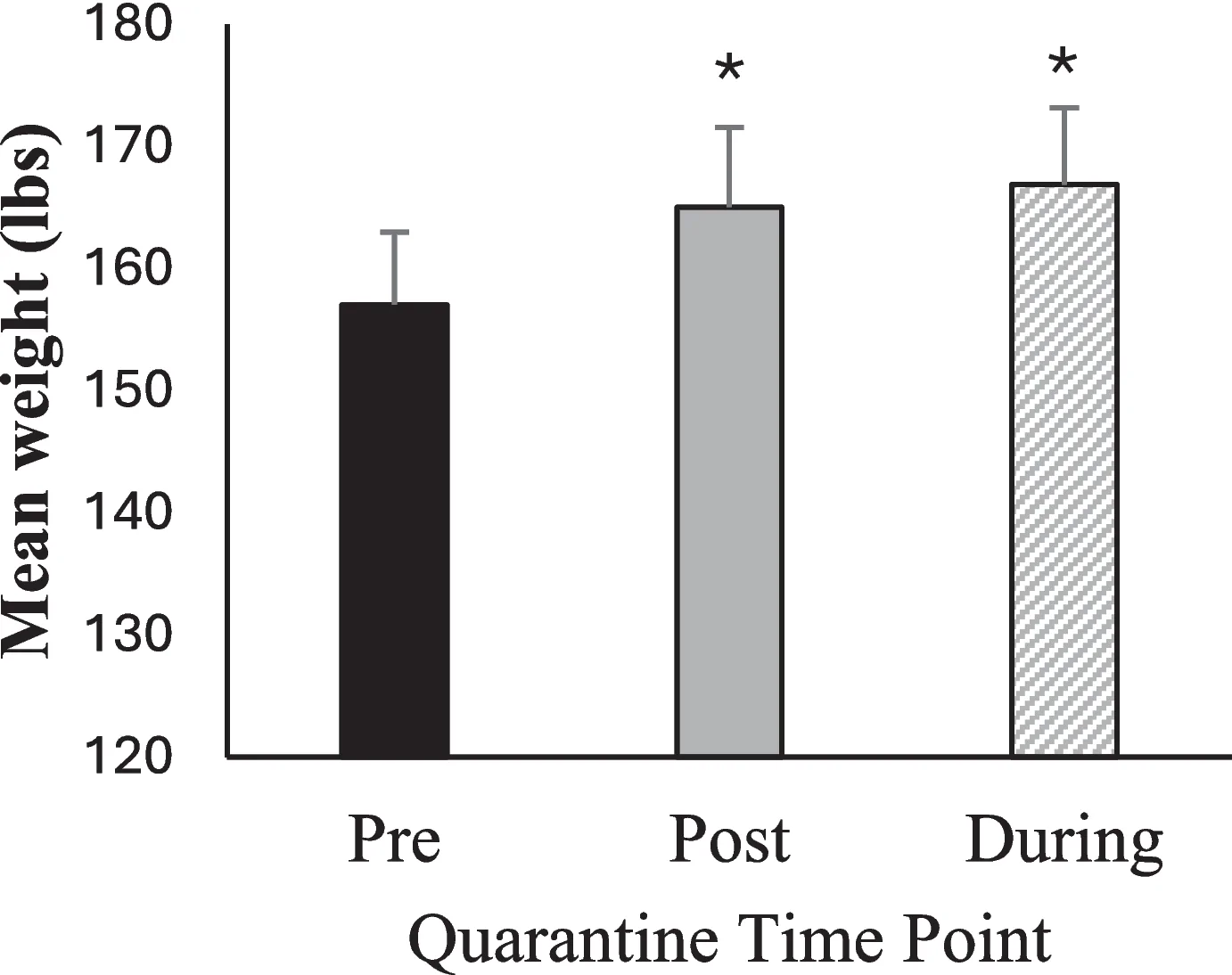
The Figure Shows Mean Self-Reported Weight (lbs.) across the Three Timepoints. *Note.* * *p* <.001 compared to baseline. *F*(49) = 13.24, *p* < .001, η2 = 0.35. Error bars = 1 SEM

In our sample of college students, we also saw self-reported weight increases over the quarantine period. Figure 5 shows self-reported weight across the three quarantine timepoints. Weight significantly increased across the three timepoints; in particular, weight rose during quarantine (*p* < 0.001) compared to the pre-quarantine timepoint and remained higher post-quarantine. This increase of approximate 7 pounds over the 3-month quarantine is within the range of other studies referenced—approximately 2.3 pounds per month (Lin et al., 2021).

In summary, the COVID-19 quarantine was associated with increases in mental health risks, such as depressive and anxious symptoms. Although the pandemic itself likely contributed to some of the variation of these symptoms, there is also support that higher consumption of a number of highly available reinforcers, including social media use, sleep, substances, and food, was also associated with these mental health endpoints. In addition, individuals more prone to overconsumption of substances and food were especially vulnerable to health risks such as drug overdose and greater weight gain, respectively.

## Discussion

The purpose of this article was to begin assessing the relations between a major, though temporary, cultural shift in accessibility to reinforcement by using a behavioral economic theoretical lens. Using peer-reviewed studies from public health, clinical psychology, and behavior analysis, as well as some data from undergraduate participants, we described changes in the consumption of some key reinforcers during the COVID-19 pandemic quarantine of 2020, and their associations with mental health and health changes. Although this was certainly not an experimental analysis of the effects of quarantine, which would have been impossible given the nature of the abruptness of the pandemic, we believe the research presented compellingly points to several important observations. First, the quarantine was associated with increased consumption of highly accessible key reinforcers, namely, social media, sleep, food, alcohol, and substances. Social media usage indeed rose, which was associated with mental health risks, such as depressive and anxious symptoms for some (Cauberghe et al., 2021; see Marciano et al., 2022, for review), but served as a protective factor for others (Gabbiadini et al., 2020; Pancani et al., 2021). In addition, sleep duration rose, and for adults, this was also associated with poor sleep quality and higher levels of mental distress (Casagrande et al., 2020; Fong et al., 2023; Marielli et al., 2021; Ranjbar et al., 2021; Sañudo et al., 2020). Consumption of alcohol and substances also increased, and predicted greater mental health and health problems for some individuals, but especially those with preexisting alcohol and substance use patterns, respectively (Almandoz et al., 2021; Chacon et al., 2021; Castaldelli-Maia et al., 2021; Grossman et al., 2020). Finally, food consumption increased and was associated with poorer diet quality and weight gain, especially with those already experiencing challenges with healthy eating (Ederer et al., 2023; Jafri et al., 2021; Jehi et al., 2023; Lange et al., 2021; Lin et al., 2021; Pellegrini et al., 2020; Salazar-Fernández et al., 2021).

Second, data from our study suggest that the higher consumption of these key reinforcers was likely related to their greater accessibility during quarantine, but was also due to lower accessibility of putative “nonessential” reinforcers, such as public entertainment, closures of in-person schools, extracurricular school activities, travel restrictions, and limitation of in-person socialization. It is important to note that these two trends in higher consumption of highly accessible reinforcers and lowered consumption of less accessible reinforcers were likely related to *a net reduction in reinforcer diversity*. The research trends and our data also suggest that these behavioral patterns returned to pre-quarantine levels after quarantine was over (though our data on substance use was limited to a low number of individuals). Third, and particularly important, this net reduction in reinforcer diversity during quarantine was associated with an elevation of mental health and health challenges for some individuals.

Before moving to the implications of this research, we want to be clear that the quarantine was a measure supported by science**.** Indeed, the data clearly show that the quarantine reduced the spread of COVID-19 and therefore saved lives (e.g., Allcott et al., 2025). However, many were unprepared by this event and were likely focused on more immediate priorities for adaptation to stay-at-home orders, such as transferring to online employment or school, ensuring they had access to food, and confirming they had accurate information on COVID-19 and measures to reduce physical health risks. As such, protections for mental health and health (other than not contracting the virus) were less of a priority.

## Implications and Recommendations

There are at least two implications of what may be learned from the COVID-19 quarantine and its effects on reinforcement diversity. First, it is possible to plan more carefully for cultural changes that may predict relatively lower reinforcer diversity and engineer environments with purpose. Although basic needs for survival, including food, water, and first aid, are usually strongly considered during times of catastrophe (and rightfully so!), the reality is that catastrophes that call for these types of preparations are rarer in most parts of the world. A more likely situation in which disruption occurs (say, for another quarantine, which is not an unlikely event; see Tognotti, 2013) may call for plans that consider reinforcement diversity and their health implications. This seems especially true for individuals who might be more prone to mental health and health risks associated with consumption of some of the key reinforcers mentioned.

Plans, for instance, may include establishing a priori rules and contingencies about family or individualized social media use that specify enhancing connectedness and discourage unhealthier use behaviors such as doomscrolling. In addition, adherence to individual or family sleep hygiene plans that include going to bed and waking at reasonable hours may reduce the odds of oversleeping or delayed sleeping, thereby better preserving sleep quality. It may also be wise to implement contingencies that maximize healthier food accessibility during times of lower reinforcer diversity; this can be done by increasing greater amounts of healthier foods in the household (if available) and minimizing consumption of higher sugar and ultra-processed food, alcohol, and substances. The maximization of plentiful and varied alternative activities and resources would also be important to increase reinforcer diversity within the home. For instance, creating space and infrastructure for high accessibility to indoor interactive entertainment (e.g., art projects, jigsaw puzzles, books, board games), outdoor sports or yard games with those within the same household (e.g., pickleball, tennis, cornhole, Spikeball, badminton), or making use of outdoor spaces with fewer people, such as public lands, would be useful. In Idaho, access to public lands is higher than most states. Some families spend a great deal of time hiking in the mountains or kayaking or canoeing in remote lakes and rivers. Fresh air and physical activity seem to enhance reinforcement processes. Indeed, individuals who have strong connectedness to nature have higher eudaimonic (purpose and meaning-based) well-being, as opposed to more hedonic well-being (see meta-analysis by Pritchard et al., 2020).

It is important to note that there are other individualized contexts in which reinforcer diversity may be lower or suddenly reduced, and these may be more likely to occur compared to a pandemic-induced quarantine. Individuals who experience abrupt hospitalization or institutionalization would also be prone to the mental health and health effects of rapid reductions in reinforcer diversity. Arranging visitations from cherished individuals could be beneficial in these situations. Moreover, these visits could include interactive activities that consider not only the preferences of the individual experiencing the change in surroundings, but also their physical, social, and cognitive needs. Family members, caregivers, parents, and friends may assist in the arrangement of greater accessibility to reinforcers, enhancing reinforcer diversity as much as possible.

Individuals who are incarcerated also experience an abrupt reduction in reinforcement diversity when they are moved from a society replete with reinforcement to one in which they spend most time in a prison cell. Because this transition is associated with mental health problems or may aggravate preexisting mental health challenges (Graham et al., 2015; Senior & Shaw, 2007; Viggiani, 2007), many scholars have called for enrichment of prison systems to include alternative reinforcers such as libraries, gyms, and group psychotherapy (e.g., see meta-analysis by Bozick et al., 2018). We see this as a suggestion for enhancing reinforcer diversity. In the United States, for instance, all federal prisons have leisure and law libraries; however, it is not clear to what extent these resources are accessed (Hussain et al., 2022). In many prisons, literacy rates are lower than the general public. For instance, in Australia about 59%–62% of incarcerated individuals have functional impairment in literacy (see Dawe, 2007; Glass, 2014), which may reduce the reinforcing potency of library offerings. This is a different kind of inaccessibility—the potential reinforcers are there, but there are literacy barriers. The same is true for physical activity opportunities. Individuals who are incarcerated are not as likely to meet recommended physical activity guidelines compared to those who are not in prison (Herbert et al., 2012). In the Scottish Prison System, for example, about half (48%) of incarcerated individuals report that they do not use prison gyms at all, despite their availability (Scottish Prison System, 2017). Designing programs that enhance accessibility to these reinforcers is important. An excellent example of this is the Fit for Life program, which is specifically designed to expose incarcerated individuals to a wide range of behavioral, physical, and mental health trainings and improvements, including group activities in which movement and physical activity are combined with establishing social connections (e.g., team sports; MacLean et al., 2022). This program not only raised physical activity participation to nearly 70% of the incarcerated population, but also increased health outcomes and self-reported enjoyment of the program.

A second implication for this article’s thesis concerns the term *reinforcer diversity.* It is our contention that this term may have some utility for situations in which abrupt changes in access to reinforcement occur, such as a pandemic or others previously mentioned. We also suggest it may be a variable that is predictive of mental health. Given the research and data presented in this article, we propose that there is a window of optimal reinforcer diversity. It is likely individualized; in that it is wider for some individuals and less so for others. It is also dynamic, in that it changes with contextual situations. This window is based on the ideal free distribution of behavior (a la Fretwell, 1969) to available valued reinforcers. Although there is room for variation in this window, the limits of what is optimal for an individual is determined *by an absence of mental health problems or dysfunction in one’s life and one’s subjective level of well-being*. This idea is an extension of what Rasmussen et al. (2020) wrote about regarding “psychological well-being” in captive animals, which they describe as both an absence of problematic behavior (with nonhumans, this would include stereotypical behavior, inactivity, and aggression), but also *the ability to allocate behavior to a range of reinforcers*.

For instance, consider the behavior and reinforcer diversity of three different graduate students in a typical 24-hr period. Student A makes progress in their program by engaging in “graduate student behavior” (e.g., studying, coursework, conducting research, teaching) 8 hr per day in this subjectively highly valued response class that produces academic reinforcement. They also make time to engage daily in other highly valued nonacademic reinforcers: exercising (1 hr), socializing in person with friends (2 hr), reading some nonprogram-related fiction that they enjoy (1 hr), streaming a valued TV show (1 hr), social media use for connective purposes (2 hr), cooking and eating healthy meals (1 hr), and prioritizing 8 hr of sleep. They generally feel subjectively well and, although stressed at times, there is an absence of chronic anxiety and depression. This is optimal reinforcer diversity for this individual.

Now consider Student B, who spends most of their time engaging in behaviors leading to academic reinforcement (16 hr/day). But, they forgo time with friends, reading for fun, exercise, sleep, social media, and they eat unhealthy food sporadically (often while in front of the computer) because of time limitations. Their high behavioral allocation to academic reinforcers likely impresses their mentors and professors, making it more likely that they will be competitive for more academic opportunities and reinforcers. In this situation, however, Student B may feel exhausted, socially isolated, and perhaps subjectively depressed or anxious, and they may not know why. This is a situation of low reinforcement diversity and note that it is overconsumption of academic reinforcement. If this high level of consumption of academic work and the associated mental health symptoms were instead related to drug consumption, most people would advise this person to seek help. This is not an optimal situation health-wise or mental health-wise for a graduate student, even though the culture of academics often reinforces this type of academic overconsumption (see Bullock et al., 2017; Evans et al., 2018). If this student sought professional help, a therapist may recommend reducing time with academics and enriching their lives with other nonacademic sources of reinforcement, i.e., to diversify their reinforcement profile (though they would probably not use these terms).

Consider also Student C, in which reinforcer diversity may be high, but their mental health is suffering. They are engaged in academic reinforcers by going to school full-time and making progress on studying and program milestones (5 hr/day). They are also engaged in full-time employment (8 hr/day) because they have a partner (with whom they spend 30 min/day) and a child (with whom they spend 2–3 hr/day). Their sleep varies (4–6 hr), but they typically awaken at 4:00 am to study because it is the only time it is quiet in the house to study. Therefore, a full night’s sleep is sacrificed. They are stressed and anxious much of the time, which is fueled by their lack of quality sleep. Their partner is frustrated and feeling neglected, and they feel they are not spending enough time with their child, leading to feelings of guilt. They are exhausted and not able to make time for self-care, such as eating healthy meals, exercising, or sleeping a typical amount. As a result, this student gets sick more often, leading to missed work and school, which leads to financial distress and difficulties keeping up academically. This, too, is not optimal reinforcement diversity. This is a different type of “burnout,” in which the student is spread too thin among too many highly valued reinforcers (Evans et al., 2018; McDermott et al., 2021). This is a situation when a person might be encouraged to “take something off their plate” or to reduce the number of behavioral commitments they have made.

We recommend that researchers who study individual, social, and cultural behavioral phenomena (i.e., metacontingencies; see Glenn, 2004) that involve shifts in reinforcer diversity—especially those related to mental health—consider assessing reinforcer diversity in their studies. There are no studies to date that specifically quantify reinforcer diversity, but there are some measures that may be suitable. One mentioned previously is the Pleasant Events Schedule (PES; MacPhillamy & Lewinsohn, 1974, 1982). The PES has been evaluated for reliability and validated with a number of special populations, including those with depression (Bouman & Luteijn, 1986), substance use (Etten et al., 1998; Khoddam et al., 2018), dementia (LeBlanc et al., 2008), Alzheimer’s disease (Logsdon & Teri, 1997), and those in long-term care (Meeks et al., 2009). A measure of environmental enrichment is another possibility (Flores-Ramos et al., 2022). This measure comprises a battery of three previously established self-report scales of physical activity, social integration, and cognitive activity (e.g., listening to music, writing a text). Scores on this battery inversely predict depression (Flores-Ramos et al., 2022), though this self-report tool has not yet been extended to other contexts of potentially low reinforcer diversity. There is also the self-report measure we use in the current article (Everyday Tasks Over COVID-19; see Appendix), which is another possibility, as items on it were selected from the Pleasant Events Scale and the environmental enrichment measure (Flores-Ramos et al., 2022). Though this measure was specifically developed for college students during the COVID-19 quarantine, items could be modified for other purposes and populations. The Everyday Tasks measure would require reliability and validation studies, as well.

Similar to the data presented in this article, there is also a need for assessing reinforcer diversity shifts in a pre-/post-manner with events that may change reinforcer accessibility abruptly, such as comparing reinforcer diversity before and after institutionalization or hospitalization. Other contexts might include cultural phenomena, such as a natural disaster or another quarantine. Developmental milestones may also be contexts to consider, such as transitioning into phases of life in which access to reinforcement may change, for instance, retirement or experiencing “empty nest” syndrome. Indeed, behavioral transitions into states that differ in reinforcer diversity are not uncommon and can be predictive of mental health behaviors. As such, we believe it requires investigation.

## Data Availability

Data from this study can be accessed through Mandalay at the following link: <To be provided on publication>

## Appendix

### Everyday Tasks During COVID-19 Quarantine

The following questions ask about some of the everyday tasks and events in a college student’s life. As you answer these questions, think about how your life was DURING (Before, After) COVID-19 QUARANTINE. Consider how you allocated your time to each task or event relative to other tasks or events. Please click on a number (0= not time, no value; 6 most time, high value) that best matches your assessments.

1. **a. During COVID-19 quarantine**, about how much time in general did you spend in school-related activities (e.g., classes, studying, extracurricular activities on campus, etc.) per week?
  **b. During COVID-19 quarantine**, how much would you say you valued school-related activities overall.
2. ** a. During COVID-19 quarantine**, about how much time in general did you spend with your spouse, significant other (i.e., boyfriend/girlfriend), or partner per week?
  **b. During COVID-19 quarantine**, how much would you say you valued your spouse, significant other, or partner overall.
3. **a. During COVID-19 quarantine**, about how much time in general did you spend per week at your place of employment?
  **b. During COVID-19 quarantine**, how much would you say you valued your place of employment overall.
4. **a. During COVID-19 quarantine**, about how much time in general did you spend with family (e.g., parents, siblings, extended family) per week?
  **b. During COVID-19 quarantine**, how much would you say you valued family overall.
5. **a. During COVID-19 quarantine**, about how much time in general did you spend in bars or clubs per week?
  **b. During COVID-19 quarantine**, how much would you say you valued bars or clubs overall.
6. **a. During COVID-19 quarantine**, about how much time in general did you spend with friends per week?
  **b. During COVID-19 quarantine**, how much would you say you valued friends overall.
7. **a. During COVID-19 quarantine**, about how much time in general did you spend in public entertainment settings (e.g., movie theaters, concerts, plays, etc.) per week?
  **b. During COVID-19 quarantine**, how much would you say you valued public entertainment settings overall.
8. **a. During COVID-19 quarantine**, about how much time in general did you spend on social media (e.g., Snapchat, Instagram, Facebook, etc.) per week?
  **b. During COVID-19 quarantine**, how much would you say you valued social media overall.
9. **a. During COVID-19 quarantine**, about how much time in general did you spend exercising (e.g., gym, sports, walks, runs, hikes, recreational physical activity, etc.) per week?
  **b. During COVID-19 quarantine**, how much would you say you valued exercising overall.
10. **a. During COVID-19 quarantine**, about how much time in general did you spend traveling (e.g., road trips, domestic travel, international travel, etc.) per week?
  **b. During COVID-19 quarantine**, how much would you say you valued traveling overall.
11. ** a. During COVID-19 quarantine**, about how much time in general did you spend at church or engaged in church related activities per week?
  **b. During COVID-19 quarantine**, how much would you say you valued church related activities overall.
12. **a. During COVID-19 quarantine**, about how much time in general did you spend in outdoor related activities (hiking, walking, running, motor sports, skiing, snowshoeing, etc.) or engaged in outdoor related activities per week?
  **b. During COVID-19 quarantine**, how much would you say you valued outdoor related activities overall.
